# Fractures as a suicidal behavior risk factor: A nationwide population-based cohort study

**DOI:** 10.1097/MD.0000000000014148

**Published:** 2019-01-18

**Authors:** Chun-Hao Tsai, Wan-Ju Cheng, Chih-Hsin Muo, Tsung-Li Lin

**Affiliations:** a Department of Orthopedics, China Medical University Hospital; b School of Medicine, China Medical University; c Department of Psychiatry, China Medical University Hospital; d Department of Public Health, China Medical University; e Management Office for Health Data, China Medical University Hospital; f Department of Sports Medicine, China Medical University, Taichung, Taiwan.

**Keywords:** fracture, population-based cohort, risk, suicidal behavior

## Abstract

This study aimed to evaluate the association between fracture history and sequential risk of suicidal behavior.

A total of 82,804 patients with fractures and 82,804 control subjects without fractures were matched. The influence of fractures on the risk of suicidal behavior-related hospital visit was analyzed using a Cox proportional hazards model.

The overall adjusted hazard ratio (aHR) of suicidal behavior-related hospital visit was 2.21 in fracture cohort. The aHR declined from 2.90 at the 1-year follow-up to 2.00 after 5 years or more. In fracture patients, the risk of suicidal behavior-related hospital visit was higher at age <35 years, with comorbidities of stroke and sleep disorder. Patients with multiple fractures had a 1.69-fold risk.

Fractures are an independent risk factor for suicidal behavior. Individuals aged younger than 35 years, with comorbidities of stroke and sleep disorder, and those who have suffered multiple fractures have elevated risk of suicidal behavior among subjects in the fracture group. Furthermore, this increased risk remained even after 5 years had passed since the fracture incident.

## 1 Introduction

Suicide is a major public health in the world. There were about 800,000 people die due to suicide every year in the world, it is notable that suicide is the second leading cause of death in 15 to 29-year-olds.^[1]^ Suicidal behavior increases the devastating impact of not only on the individual at risk, but also their families, communities, and society.^[2]^

Mann et al^[3]^ proposed a Stress-Diathesis Model of suicidal behavior, in which suicidal behavior is conceptualized as the result of interaction between stress factors, such as acute mental or psychosocial stress and consequential self-reactions. Several clinical, psychosocial, and demographic factors have been shown to increase the risk of suicidal behavior. Physical diseases also contribute to late-life suicidal behavior, such as spinal fracture was found to be associated with a higher risk of suicidal behavior among older aged over 65 years without the specific disorder within 3 years.^[4]^ However, whether a previous fracture history increases the risk of suicidal behavior in general population has not yet to be studied in the literature.

Therefore, we conducted this nationwide population-based retrospective cohort study by using the database of a universal insurance program to evaluate the association between fracture history and the risk of suicidal behavior-related hospital visit.^[5]^

## 2 Methods

### 2.1 Data Source

Used the longitudinal health insurance database (LHID 2000) to establish the retrospective cohort study to investigate the association between fracture and suicidal behavior. The LHID 2000 is one of the National Health Insurance Research Database (NHIRD) in Taiwan, which contained the claim data of health care from the Taiwan National Health Insurance (NHI) program. Since 1995, a single-payer NHI program was launched by the government of Taiwan. The coverage rate was nearly 99% of the resident in Taiwan. The LHID 2000 comprises claims data collected from 1 million people randomly selected from the total insurant population from 1996 to 2013, which including information of patients such as demographic status and claims data for inpatient and outpatient care. The disease record system was according to the International Classification of Diseases, Ninth Revision, Clinical Modification (ICD-9-CM).

The NHIRD encrypts patient personal information to protect privacy and provides researchers with anonymous identification numbers. This study was approved to fulfill the condition for exemption of waiver of consent by the Institutional Review Board (IRB) of China Medical University in Taiwan (CMUH104-REC2-115 (CR-1).

## 3 Study population

Based on the ICD-9-CM codes, we defined the fracture patients by the code of 800–829 from outpatient or inpatient care. Each patient's fracture diagnosis date was defined the index date. Total numbers of 82,804 fracture patients were collected from 2000 to 2006 as the fracture cohort.

For each fracture patient, 1 comparison subject was matched for sex, age (5-year intervals), and index year form subjects without fracture diagnosis in the NHIRD (non-fracture cohort; n = 82,804) by the frequency matched method. Frequency matching was a sampling design used in case–control studies to assure that cases and controls have the same distributions over strata defined by matching factors. Frequency matching was also used in cohort studies to insure that exposed and unexposed individuals have the same distributions over strata defined by known risk factors.^[6]^

During the follow-up period subjects with occurrence of suicidal behavior-related hospital visit (ICD-9-CM, External Cause Codes [E-Codes]: 950–959) was defined as the event outcome. A suicidal behavior-related hospital visit was defined as any visit in which the patient presented with suicidal ideation or suicide attempt from outpatient or inpatient claims.^[5]^ Subjects with a history of unsuccessful suicide attempt before the index date were excluded.

Follow-up person-years were calculated for each subject from index date until December 31, 2013, through to the date of diagnosis of suicidal behavior-related hospital visit or withdrawal from the insurance system.

Those risk factors, including age, sex, and associated comorbidities, were controlled in the analysis model for adjustment. We obtained baseline comorbidities including diabetes (ICD-9-CM: 250), hypertension (ICD-9-CM: 401–405), heart failure (ICD-9-CM: 402.01, 402.11, 402.91, 404.01, 404.03, 404.11, 404.13, 404.91, 404.93, and 428.0), stroke (ICD-9-CM: 430–438), anxiety (ICD-9-CM: 300.0, 300.2, 300.3, 308.3, and 309.81), irritable bowel syndrome (IBS; ICD-9-CM: 564.1), headache (ICD-9-CM: 307.8, 339.1, and 346), sleep disorder (ICD-9-CM: 307, 327, and 780.5), and liver cirrhosis (ICD-9-CM: 571). Subjects with a history of depression (ICD-9-CM: 296.2, 296.3, 300.4, and 311), bipolar (ICD-9-CM: 296), or schizophrenia (ICD-9-CM: 295 and V11.0) were excluded, because those are strong predictors for suicidal behavior-related hospital visit, and not be considered as comorbidity. To validate comorbidity, people with at least 3 times outpatient, or once inpatient care for comorbidity were defined as patients.

Four different fracture localization subgroups are separated by ICD-9-CM codes from fracture cohort including trunk (ICD-9-CM: 805–809), upper limbs (ICD-9-CM: 810–819), lower limbs (ICD-9-CM: 820–829), and multiple fracture.

## 4 Statistical analyses

The SAS 9.4 software (SAS Institute, Cary, NC) was used for data management and statistical analyses. Two-sided *P* value of <.05 was considered significant. Using the chi-square test to assay the difference in those categorical variables between non-fracture and fracture cohorts, including sex, age-subgroup (<35, 35–65, and ≥65 years), and each comorbidity history (no/yes).

The mean and standard deviation (SD) of age was analyzed using the Student *t* test. The incidence density of suicidal behavior was calculated in non-fracture and fracture cohorts, which as the sum of suicidal behavior-related hospital visit events divided by the sum of observation time (per 10,000 person-years). In 2 cohorts, the crude hazard ratios (HRs) for suicidal behavior-related hospital visit and their 95% confidence intervals (95% CIs) were measured for these variables by using the Cox proportional hazards models. Compared with the non-fracture cohort, the adjusted HRs (aHRs) of suicidal behavior-related hospital visit was measured in fracture cohort by the multivariable Cox proportional hazards model after adjusted for age, sex, and comorbidity (including diabetes, hypertension, heart failure, stroke, anxiety, IBS, headache, sleep disorder, and liver cirrhosis). Follow-up time stratified analysis was presented the risk of suicidal behavior-related hospital visit in fracture cohorts compared with non-fracture cohort. We also estimated the risk factors of suicidal behavior-related hospital visit in fracture patients by multivariable Cox model. The association between suicidal behavior-related hospital visit and fracture stratified by metal-associated disease stratified analysis was assessed. The cumulative suicidal behavior incidence curves for the both cohorts were plotted using the Kaplan–Meier method, and the differences between the curves were analyzed using the log-rank test.

## 5 Results

Total of 82,804 patients with fracture and 82,804 patients without fracture were included in our study (Table 1). The mean age of our participants was 41.4 years (SD: 22.6). After frequency matching method, we determined no significant differences of sex and age between the fracture and non-fracture cohorts. Both cohorts were predominantly men (56.1%) and <35 years of age (42.6%).

**Table 1:**
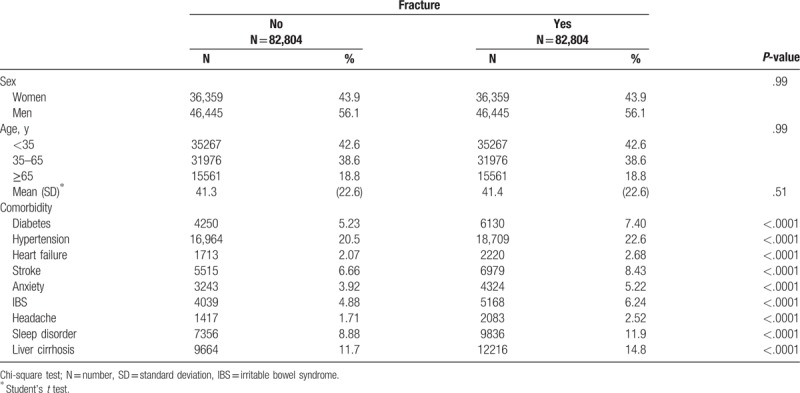
Comparison of demographics and each comorbidity history in our study population.

**Figure 1:**
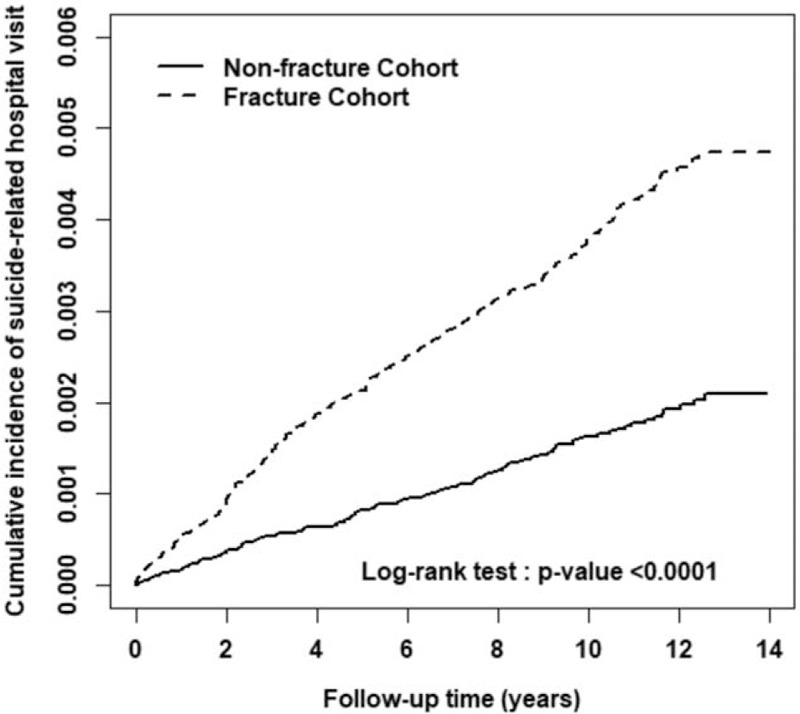
The cumulative incidence of suicidal behavior-related hospital visit in fracture cohort (dashed line) and non-fracture cohort (solid line).

The cumulative incidence of suicidal behavior-related hospital visit in the fracture cohort was significantly higher than that in the non-fracture cohort (*P*-value <.0001, log-rank test; Fig. 1).

The overall crude risk of suicidal behavior-related hospital visit in the fracture cohort was 2.37-fold higher than that in the non-fracture cohort (95% CI: 1.93–2.91; Table 2). Although significant greater number of comorbidities of fracture cohort (Table 1) may have been predisposed to suicide,^[4]^ however, after adjusting for those risk factors (including age, sex, and each comorbidity history), the aHR of suicidal behavior-related hospital visit was presented 2.21 in fracture cohort (95% CI: 1.80–2.71). Stratified analyses by follow-up year using multivariable Cox model, the hazard ratio of suicidal behavior-related hospital visit in fracture cohort compared with the non-fracture group declined slightly with time. The aHR declined from 2.90 (95% CI: 1.58–5.32) at the 1-year follow-up to 2.00 (95% CI: 1.49–2.70) after 5 years or more, which suicidal behavior-related hospital visit risk was still high even after time goes by (Table 2).

**Table 2:**
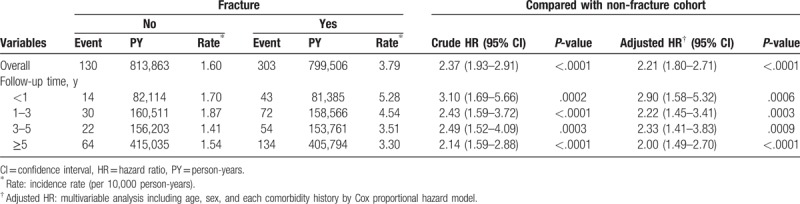
Incidence of suicidal behavior-related hospital visit between fracture and non-fracture groups stratified by follow-up year.

Table 3 presents the risk factors of suicidal behavior-related hospital visit in fracture patient. In multivariable Cox model, patients at age <35 years had a higher risk than those at ≥65 (aHR: 2.71, 95% CI: 1.99–3.67), with stroke than those without stroke (aHR: 1.71, 95% CI: 1.14–2.58), with sleep disorder than those without sleep disorder (aHR: 1.84, 95% CI: 1.33–2.56), multiple fracture patients than upper limbs (aHR: 1.69, 95% CI: 1.17–2.43).

**Table 3:**
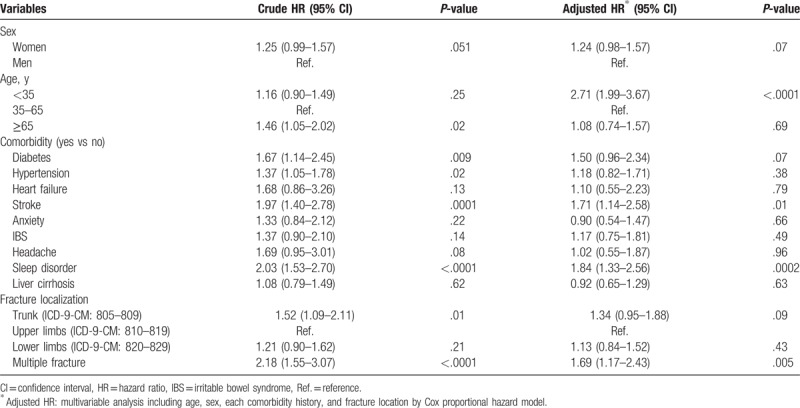
The risk factor of suicidal behavior-related hospital visit in fracture cohort (n = 88,689).

## 6 Discussion

The current study demonstrated that fracture history could lead to an increased risk of suicidal behavior. The risk of suicidal behavior was still high even after 5 years from fracture. Individuals aged younger than 35 years, with comorbidities of stroke and sleep disorder, and those who have suffered multiple fractures have elevated risk of suicidal behavior among subjects in the fracture group.

This nationwide population-based retrospective cohort study in Taiwan found a previous fracture history increases the risk of suicidal behavior, and our finding was similar as the nationwide, register-based cohort study in Denmark.^[4]^

Various mechanisms after fracture may account for an increased risk of suicidal behavior. Possible biological processes associated with fracture-induced inflammation mediate increased risk of suicidal behavior. Fractures increase serum systemic inflammatory response syndrome-related cytokines, such as Interleukin 6 (IL-6),^[7]^ which maintain high levels of bone fracture for several months to induce bone remodeling.^[8,9]^ IL-6 is a key factor in the pathophysiology of post-fracture depression.^[10]^ Elevated IL-6 changes brain function and can promote suicidal behavior, especially suicidal ideation and non-fatal suicide attempts.^[10]^ The related postmortem study found differences in messenger ribonucleic acid expression of IL-6 in the hippocampus of hippocampal samples collected from individuals after suicide deaths.^[11]^ In addition, the meta-analysis yielded results on cytokines and suicidal behaviors: the most consistent finding was elevated IL-6 in cerebrospinal fluid, blood, and postmortem brain.^[12]^ External factors may increase intracellular brain cytokine production, such as fracture-related stress-induced microglia activation.^[13]^ Moreover, microglial cells are more directly involved in suicidal behavior risk.^[14]^ Postmortem studies have shown that, regardless of their primary psychiatric diagnosis, patients who die from suicidal behavior exhibit increased microglial levels as markers of neuroinflammation.^[15]^

Psychosocial stress after fractures in young patients is a major contributor to advanced suicidal behavior.^[16]^ Early life stress and abnormal cortisol stress affect sustained high levels of pro-inflammatory cytokines including tumor necrosis factor-alpha (TNF-α) and IL-6, which cause an increased inflammatory response after psychosocial stress.^[10,17]^ In addition, young patients who experience fractures may suffer chronic pain and systemic inflammation over time; therefore, these individuals may exhibit early deterioration of their physical condition. Consequently, this finding may partly explain why we found a higher risk of suicidal behavior-related hospital visit in young patients (<35 years) with fractures.

Stroke can cause limb disability and physical impairment, and is one of the most debilitating neurological disorders.^[18]^ Stroke increased the risk for suicidal ideation and attempt.^[19,20]^ Fracture in post-stroke survivors may deteriorate the impaired function and worsen the quality of life. This finding may explain the higher risk of suicidal behavior-related hospital visit in fracture patients with history of stroke.

After an acute orthopedic injury, some patients suffered from posttraumatic stress disorder (PTSD), especially lower extremity fracture and multiple extremity fractures.^[21,22]^ Sleep disorder is the hallmark of PTSD and may persist for years after traumatic events.^[23]^ Chronic pain after fracture can complicate the management of sleep disorders and worsen sleep quality, and was found to be predictors of suicidality.^[24,25]^ A lot of evidence shows sleep disorder, including insomnia, nightmares, and sleep insufficiency, is associated with enhanced suicidal risk.^[26–29]^ This finding may explain why patients with comorbidity of sleep disorder after fracture were significantly related to the occurrence of suicidal behavior-related hospital visit.

In current study, we found higher risk of suicidal behavior-related hospital visit in patients after multiple fracture. Long-term physical disability, functional decline, and impaired quality of life after multiple fracture may result in increased dependence on help, sensory burden, and disconnection from social networks, which in turn are contributing factors to suicidal behavior.^[15,30,31]^ Moreover, chronic pain and prolonged systemic inflammation were also important risk factors for suicidal behavior after suffering from multiple fracture.^[25,32,33]^

Each factor and mechanism may have different effects over time, which explains why we revealed an increased risk of suicidal behavior even after >5 years had passed since the fracture's occurrence.

This study had several limitations. First, the NHIRD does not contain detailed information on lifestyle, educational status, living conditions, or pre-injury mental state, which could all affect suicidal behavior risk. Second, all data in the NHIRD are confidential; therefore, relevant clinical variables such as fracture severity, surgical methods, and serum laboratory data were not available for analysis. Third, evidence from retrospective cohort studies is generally statistically lower than the evidence from randomized trials because of the potential bias associated with adjusting the confounding variables. Lastly, we could not recruit those with suicidal ideologies or tendencies or others who did not seek medical attention.

Despite these limitations, the main advantage of this study is the use of population-based data, which is highly representative of the general population.

## 7 Conclusion

Fractures are an independent risk factor for suicidal behavior. Individuals aged younger than 35 years, with comorbidities of stroke and sleep disorder, and those who have suffered multiple fractures have elevated risk of suicidal behavior among subjects in the fracture group. Furthermore, this increased risk remained even after 5 years had passed since the fracture incident. The medical system should aware fracture is the risk of suicidal behavior and early interventions to prevent suicidal behavior after fracture by multidisciplinary and comprise medical, social, physical, and psychological strategies.

## Author contributions

All authors made substantive intellectual contributions to this study to qualify as authors. Tsung-Li Lin and Chun-Hao Tsai designed the study. Chih-Hsin Muo and Tsung-Li Lin collected subjects “data.” Chih-Hsin Muo performed statistical analysis. An initial draft of the manuscript was written by Tsung-Li Lin. Wan-Ju Cheng and Chun-Hao Tsai re-drafted parts of the manuscript and provided helpful advice on the final revision. All authors were involved in writing the manuscript. All authors have read and approved the final manuscript.

**Conceptualization:** Chun-Hao Tsai.

**Data curation:** Wan-Ju Cheng, Chih-Hsin Muo.

**Formal analysis:** Chih-Hsin Muo.

**Methodology:** Wan-Ju Cheng, Chih-Hsin Muo.

**Resources:** Chun-Hao Tsai.

**Supervision:** Tsung-Li Lin.

**Visualization:** Wan-Ju Cheng.

**Writing – original draft:** Tsung-Li Lin.

**Writing – review & editing:** Chun-Hao Tsai.
